# The effects of silver nanoparticles biosynthesized using fig and olive extracts on cutaneous *leishmaniasis*-induced inflammation in female balb/c mice

**DOI:** 10.1042/BSR20202672

**Published:** 2020-12-16

**Authors:** Mina A. Almayouf, Manal El-khadragy, Manal A. Awad, Ebtesam M. Alolayan

**Affiliations:** 1 Faculty of Science, Zoology Department, King Saud University, Riyadh 11451, Saudi Arabia; 2 Biology Department, Faculty of Science, Princess Nourah Bint Abdulrahman University, Riyadh 11671, Saudi Arabia; 3 Department of Zoology and Entomology, Faculty of Science, Helwan University, Cairo 11795, Egypt; 4 King Abdullah Institute for Nanotechnology, King Saud University, Riyadh 11451, Saudi Arabia

**Keywords:** antioxidant, Cutaneous leishmaniasis, Fig, gene expression, Olive, Silver nanoparticles

## Abstract

*Leishmaniasis* is a group of infectious and noncontagious severe parasitic diseases, caused by protozoans of the *Leishmania* genus. Natural products characterize a rich source of prospective chemical entities for the development of new effective drugs for neglected diseases. Scientific evaluation of medicinal plants has made it possible to use some metabolites from flavonoids and polyphenols compounds for the treatment of parasitic diseases. Therefore, we aimed in the present study to evaluate the protective effect of silver nanoparticles (Ag-NPs) biosynthesized using Fig and Olive extracts (NFO) against Cutaneous *leishmaniasis* in female Balb/c mice. A total of 70 mice were used and divided into seven groups. Treatment was initiated when local lesions were apparent, we found that Fig and Olive extracts were found to be a good source for the synthesis of (Ag-NPs), their formation was confirmed by color change and stability in solution. Nanoparticles biosynthesized using Fig and Olive extracts induced a reduction in the average size of cutaneous leishmaniasis lesions compared with the untreated mice. Moreover, nanoparticles treatment decreased oxidative stress (LPO, NO), down-regulation gene expression levels (*TNF-α, IL-1β*, and *BAX*), and this antileishmanial activity of nanoparticles was associated with enhanced antioxidant enzyme activities. In addition, histopathological evaluation proved the antileishmanial activity of nanoparticles compared with the positive control.

Therefore, we aimed in the present study to evaluate the protective effect of silver nanoparticles biosynthesized using Fig and Olive extracts against cutaneous lesions induced by Leishmania *major* infection through their anti-inflammatory, antioxidant activities, and faster clinical efficacy than standard pentavalent antimonial treatment.

## Introduction

*Leishmaniasis* is a parasitic disease caused by infection with protozoan parasites of the genus *Leishmania*, which spread by biting of infected female sand flies [1]. The genus *Phlebotomus* is considered the vector of *leishmaniasis* [2]. Several forms of *leishmaniasis* have been detected including Cutaneous *leishmaniasis* (CL), Mucocutaneous *leishmaniasis* (MCL), and Visceral *leishmaniasis* (VL) [3].

CL spreads all over the Kingdom of Saudi Arabia with greatly varying prevalence rates in different regions. According to the Ministry of Health statistical yearbook, in 2018, Al-Qassim region presented the highest percentage of the total number of cases (17.4%), followed by Al-Medinah (16.6%), and then Al-Ahsa (13.4%). CL lesions usually resolve spontaneously in a few months (without treatment). However, a disfiguring scar may remain at the infection site due to slow healing and may lead to self-stigma and social stigma and have negative psychological effects on the young [4]. The currently used pentavalent antimony drugs, such as pentamidine and amphotericin B, may display toxicity, and have also unpleasant side effects. Furthermore, parasite may develop resistance to treatment. Therefore, the lack of vaccines, the emergence of drug resistance and the adverse effects of currently used drugs emphasize the need to discover novel drugs, particularly from natural products. Efforts to find alternative cost effective, nontoxic drugs against *leishmaniasis* have led to the development of a few potential drugs from natural resources [5].

The use of natural products as combinations (blends) is a common practice in Saudi Arabia and has existed in many cultural systems for centuries [6]. Natural products provide prototypes for pharmacologically active compounds, particularly as antimicrobial, anticancer, and antiparasitic agents. Several internal or external pathological factors such as parasitic infections interrupt the oxidant/antioxidant balance, leading to oxidative stress. The antioxidant defense system is closely connected with nutrition [7,8].

Synthesis of silver nanoparticles (Ag-NPs) utilizing fungi, bacteria, or plant extracts has emerged as an alternative approach. There are several reasons for interest in green biosynthetic methods for (Ag-NPs). They are simple, cost-effective, provide large quantities, are harmless and environmentally friendly [9]. The reduction and stabilization of silver ions is achieved by combining biomolecules such as amino acids, proteins, enzymes, alkaloids, polysaccharides, phenolics, tannins, vitamins and saponins, from plant extracts that are already established as having therapeutic value [10]. In recent years, they have attracted considerable attention for chemical and medical applications due to their exceptional properties including anti-inflammatory, antibacterial, and antiparasitic activities, high resistance to oxidation and promotion of wound healing [11,12]. Biosynthesis of (Ag-NPs) has been carried out by utilizing ethanol extracts from *Cardiospermum halicacabum* L. leaves [13], *Impatiens balsamina* L. leaves [14], and *Lantana camara* L. fruits [15]. Psoralen is a novel compound that is used to reduce pathological effects of *L. major, L. infantum*, and *L. chagasi* promastigotes. Also, Oleuropein derived from the Olive tree, *Olea europaea L*. (Oleaceae), is a biophenol with many biological activities. A previous study has shown that Oleuropein exhibits leishmanicidal effects against three *Leishmania spp. in vitro* and minimizes the parasite burden in *L. donovani*-infected Balb/c mice [16].

*Ficus carica Linn* is an Asian and Middle East species of flowering plant that belong to the mulberry family. It contains high levels of anthocyanins, polyphenols (such as Psoralen), and flavonoids, are exhibits good antioxidant capacity and pharmacological actions including antioxidant, antibacterial, anti-inflammatory, gastroprotective, vulnerary, anticancer, antispasmodic, antiparasitic, and immunobalancing activities [17–20]. The Psoralen compound amotosalen has been used to reduce the pathological effects of *L. major* inoculation and the attenuation of virulence of *L. infantum* and *L. chagasi* promastigotes [21,22].

*Olea europaea L*. (Olive), the most well-known species of the genus *Olea*, is one of the most famous fruit crops all over the Middle East [23]. According to the previous studies, different parts of *O. europaea* have been used in folk medicine to remedy some illness [24]. Moreover, its pharmacological features such as antidiabetic, anticancer, anti-inflammatory, antihypertensive, antimicrobial, and antiparasitic effects have been attributed to this plant [25,26].

Pharmacological properties of *F. carica Linn* and *Olea europaea L*. (Olive) extracts has been scrutinized, with antimicrobial, antiparasitic, anti-inflammatory, and immunobalancing effects mainly due to the presence high level of flavonoids and polyphenols compounds that possess the strongest antioxidant potential effects [22,25,26].

In the present study, Ficus carica Linn (Fig) and Olea europaea (Olive) extracts with (Ag-NPs) exhibited remarkable antileishmanial property. This activity may be attributed to the presence of flavonoids and polyphenols compounds that acts as antileishmanial potency by enhancing scavenges free radicals that produced by *L. major* and by increasing the activity of antioxidant defense system.

Hence, we examined the efficacy of silver nanoparticles biosynthesized by using Fig (*Ficus carica Linn*) and Olive (*Olea europaea*) extracts against (*L. major*) induced inflammation in female Balb/c mice.

## Materials and methods

### Plant material and extract preparation

Dried fruits of *Ficus carica* were purchased from the local market in Riyadh. About 10 g of the dried fruit was crushed, incubated for 10 min in 100 ml distilled water at 60°C and filtered through Whatman no.1 filter paper (pore size 25 mm). The filtrate was further filtered through 0.6 mm sized filters. The resulting filtrate was immediately used for preparing (Ag-NPs) as previously described [19].

Fresh leaves of *Olea europaea* were collected from the Alhamedih farm owned by Almayouf Abdulhamid in Sakaka Aljouf, surface cleaned with running tap water to remove debris and other contaminated organic contents, followed by double distilled water and air dried at room temperature. About 10 g of crushed dried leaves were kept in a beaker containing 100 ml distilled water and incubated for 10 min at 60°C. The extract was cooled down and filtered with Whatman filter paper no.1 and the resulting extract was immediately used for preparing (Ag-NPs) as previously described [27].

### Phenolic content

Total phenolic compound content of dried fruits for each *Ficus carica* and dried leaves of *Olea europaea* were assayed by the Folin-Ciocalteu method as described previously [28]. Briefly, 0.1 ml of the sample’s extract was mixed with 2.5 ml of distilled water in a test tube, and then 0.1 ml of undiluted Folin-Ciocalteu reagent (Sigma-Aldrich, St. Louis, MO, U.S.A.) was added. The solution was mixed well and then allowed to stand for 6 min before adding 0.5 ml of 20% sodium carbonate solution. The color developed for 30 min at room (20°C) temperature and the absorbance was measured at 760 nm using a spectrophotometer (PD 303 UV spectrophotometer, Apel Co., Limited, Saitama, Japan). A blank sample was prepared using 0.1 ml of methanol instead of the extract. The measurement was compared to a calibration curve of gallic acid solution and expressed as milligram (mg) equivalent (eq.) of gallic acid per gram (g) of dry weight extract.

### Flavonoids

The aluminum chloride colorimetric method was used to determine the total flavonoid content for each dried fruits of *Ficus carica* and dried leaves of *Olea europaea* as described previously [29]. Briefly, in a test tube, 50 µl of the extract was mixed with 4 ml of distilled water, 0.3 ml of 5% NaNO_2_ solution, and 0.3 ml of 10% AlCl_3_.6H_2_O. The mixture was allowed to stand for 6 min and then 2 ml of 1 mol/l NaOH solution was added; distilled water was subsequently added to bring the final volume to 10 ml. The mixture was allowed to stand for another 15 min and the absorbance was measured at 510 nm. The total flavonoid content was calculated from a calibration curve and the result is expressed as mg eq. rutin per g dry weight.

### DPPH (2,2–diphenyl–1–picrylhydrazyl) radical scavenging activity

The power of the of dried fruits for each *Ficus carica* and dried leaves of *Olea europaea* to scavenge DPPH radicals was assayed as described previously [30]. A fresh solution of 0.08 mM DPPH radical in methanol was prepared. Next, 950 µl of DPPH solution was mixed with 50 µl extract and incubated for 5 min. Exactly 5 min later, the absorbance of the mixture was measured at 515 nm (PD 303 UV spectrophotometer, Apel Co., Limited). Antioxidant activity (AA) is expressed as percentage inhibition of DPPH radical using the equation below; AA = 100 − [100 × (A sample/A control)], where A sample is the absorbance of the sample at time, *t* = 5 min and A control is the absorbance of the control.

### ABTS [2,4,6–tri(2–pyridyl)–s–triazine] radical scavenging activity

The ABTS assay was used to determine the DPPH radical scavenging activity according to the method of Gouveia and Castilho (2011) [31]. The ABTS^.+^ radical solution was prepared by reacting 50 ml of 2 mM ABTS solution with 200 µl of 70 mM potassium persulfate solution. This mixture was stored in the dark for 16 h at room temperature and it was stable in this form for 2 days. For each analysis, the ABTS^·+^ solution was diluted with pH 7.4 phosphate-buffered saline (PBS) solution to an initial absorbance of 0.700 ± 0.021 at 734 nm.

This solution was freshly prepared for each set of analysis. To determine the antiradical scavenging activity, an aliquot of 100 µl methanolic solution was mixed with 1.8 ml of ABTS^.+^ solution, and the decrease in absorbance at 734 nm (PD 303 UV spectrophotometer, Apel Co., Limited, Saitama, Japan) was recorded during 6 min. The results are expressed as µmol Trolox equivalent per g of dried extract (µmol eq. Trolox/g), based on the Trolox calibration curve.

### Ferric reducing antioxidant power (FRAP)

Ferric reducing antioxidant power (FRAP) was performed as described previously [32]. The FRAP reagent included 300 mM acetate buffer, pH 3.6, 10 mM 2,4,6–Tris(2–pyridyl)–s–triazine (TPTZ) in 40 mM HCl, and 20 mM FeCl_3_ in the ratio 10:1:1 (v/v/v). A volume of 3 ml of the FRAP reagent was mixed with 100 µl of each dried fruits of *Ficus carica* and dried leaves of *Olea europaea* in a test tube and incubated with shacking at 37°C for 30 min in a water bath. Reduction of ferric–TPTZ to the ferrous complex formed an intense blue color, which was measured with a UV–visible spectrophotometer (PD 303 UV spectrophotometer, Apel Co., Limited) at 593 nm after 4 min. The results are expressed in terms of mol eq. Trolox per g of dried sample (µmol eq. Trolox/g).

### Synthesis of silver nanoparticles

Green silver nanoparticles were synthesized by bioreduction of Ag^+^ using fresh suspension of Fig and Olive extracts. About 5 ml of mixed extract was added drop by drop to an aqueous solution of AgNO_3_ (50 ml, 0.1 mM/ml) and was stirred at 45–50°C for 30 min. Then, ultrasonication was applied to the mixed solution for 3 h. The colorless silver nitrate solution was changed to a deep brown solution, indicating the formation of (Ag-NPs). The residual AgNO_3_ was removed by dialysis against deionized water at 4°C. The formed (Ag-NPs) were analyzed by Zeta sizer (ZEN 3600, Malvern, U.K.) and characterized using transmission electron microscopy (TEM) (JEM-1011, JEOL, Akishima, Japan). Furthermore, the green (Ag-NPs) synthesis was confirmed by using a UV–Vis spectrophotometer in the range of 200–1000 nm wavelength. The absorption spectra were recorded with Perkin–Elemer Lambda 40 B double beam spectrophotometer using 1 cm matched quartz cells. The stability of (Ag-NPs) was examined by observing the color of the solution after 20, 40, 50, and 60 days of storage at 4°C.

### *Leishmania major* and culture

*L. major* promastigotes (MHOM/SA/84/JISH) of a Saudi substrain were maintained in RPMI1640 medium (GIBCO, New York, NY, U.S.A.) containing fetal bovine serum (FBS) (Sera Laboratories International, Horsted Keynes, U.K.), 100 U/ml penicillin + 100 mg/ml streptomycin (BioWhittaker, Verviers, Belgium), and 1% L-glutamine in 25 ml culture flasks. Each flask was incubated in an incubator at 25°C. Cultures were passaged after 4 days of incubation. Morphology and motility of promastigotes were observed by using an inverted microscope.

### Experimental protocol

Balb/c female mice (*n*=70; eight weeks old) for the *in vivo* experiment were obtained from the animal house at the Female Center for Scientific and Medical colleges, Riyadh, KSA and the animal work has taken place in the Zoology Department, College of Science, King Saud University, Riyadh, KSA. Mice were challenged with *L. major* by subcutaneous injection of 0.1 ml of RPMI1640 media containing 10^7^ promastigotes [33]. The animals were housed in wire-bottomed cages under standard conditions of illumination with a 12-h light–dark cycle and at a temperature of 25 ± 1°C for one week until the beginning of treatment. Animals were provided with tap water and a balanced diet ad libitum.

The animals were randomly divided into seven groups with 10 mice in each group, Group 1: (NC) Normal non-infected negative control group. Group 2: (PC) Infected untreated positive control group: Mice were subcutaneously inoculated with 1 × 10^7^ promastigotes in a shaved area above the tail [33].

Group 3: (P) Infected mice treated with sodium stibogluconate (Pentostam) (Pen; 120 mg/kg intramuscularly) for 4 weeks starting with the first appearance of an ulcerative lesion [34], Group 4: (NFOB) Mice pretreated with oral (Fig and Olive) nanosilver (0.2 mg/mouse) 2 weeks before infection [35], Group 5: (FOB) Mice pretreated with oral (Fig and Olive) (0.2 mg/mouse) 2 weeks before infection, Group 6: (NFOA) Infected mice treated with oral (Fig and Olive) nanosilver (0.2 mg/mouse) for 4 weeks starting with the first appearance of an ulcerative lesion, Group 7: (FOA) Infected mice treated with oral (Fig and Olive) (0.2 mg/mouse) for 4 weeks starting with the first appearance of an ulcerative lesion. Mortality was checked daily and parasitemia was assessed every other day by observing the appearance of lesions (2–6 weeks post-infection). Each week, the lesion size was measured before and after treatment with Vernier calipers in two diameters (a, b). The lesion size was calculated using the following formula [36]:

$$\text{Lesion Size (LS)}:\;\frac{\text{a}\;+\;\text{b}}{2}$$

Four weeks post-infection, mice were anaesthetized by CO_2_, asphyxiation then sacrificed by guillotine and skin at the side of lesion was excised promptly. Skin samples for biochemical and molecular analysis were frozen at −80°C until processed.

### Oxidative stress

Homogenates of the skin were prepared in 50 mM Tris-HCl and 300 mM sucrose to measure lipid peroxidation (LPO) in terms of the amount of malondialdehyde (MDA) formed using the thiobarbituric acid (TBA) method [37]. Whereas Green et al*.* [38] and Ellman methods [39] were applied to determine the levels of nitrite/nitrate (nitric oxide; NO).

### Enzymatic antioxidant status

The skin homogenates were also used to determine superoxide dismutase (SOD) [40], catalase (CAT) [41], and glutathione (GSH) according to the method of Ellman [39].

### Real-time PCR

Total RNA was extracted from the skin tissue samples using a RNeasy plus Minikit (Qiagen,Valencia, CA, U.S.A.). RNA was reverse transcribed using the RevertAid H minus Reverse Transcriptase (Fermentas, Thermo Fisher Scientific Inc., Waltham, MA, U.S.A.). Real time PCR reactions were performed using Applied Biosystems 7500 Instrument. The relative gene expression was determined with power SYBR Green (Life Technologies, Carlsbad, CA, U.S.A.) and by the comparative threshold cycle method of Pfaffl [42]. The PCR primers for *BAX, BCL-2, TNF-α*, and *IL-1β* genes were synthesized by Jena Bioscience GmbH (Jena, Germany). Primers were designed using the Primer-Blast program from NCBI.

mRNA levels for each sample were normalized to β-actin. The primer sets used the following:

Bax (S): 50– GTT TCA TCC AGG ATC GAG CAG –30.
Bax (AS): 50– CAT CTT CTT CCA GAT GGT GA –30.
Bcl-2 (S): 50– CCT GTG GAT GAC TGA GTA CC –30.
Bcl-2 (AS): 50– GAG ACA GCC AGG AGA AAT CA –30.
TNF-α (S): 50– AGAACTCAGCGAGGACACCAA –30.
TNF-α (AS): 50– GCTTGGTGGTTTGCTACGAC –30.
IL-1β (S): 50– GACTTCACCATGGAACCCGT –30.
IL-1β (AS): 50– GGAGACTGCCCATTCTCGAC –30.

### Determination of apoptotic markers in skin tissue

Skin sample homogenates were prepared in lysis buffer and analyzed using a Colorimetric Caspase-3 Assay Kit (Sigma-Aldrich Co., Saint Louis, MO, U.S.A.), according to the manufacturer’s instructions. Whereas, BCL-2 and BAX protein levels were determined in skin homogenates by using ELISA kits (R&D Systems Inc., Minneapolis, MN, U.S.A.), according to the manufacturer’s instructions. Levels were expressed as ng/mg tissue protein.

### Histological examination

The tissues were collected and immediately fixed with 10% buffered formalin and embedded in paraffin. Sections (4–5 μm) were prepared and then stained with hematoxylin and eosin dye for photomicroscopic observations.

### Statistical analysis

Results are represented as means ± standard deviation of the means (SD). Data were analyzed by one-way analysis of variance (ANOVA). For the comparison of significance between groups, Duncan’s test was used as post hoc test according to the Statistical Package for the Social Sciences (SPSS version 20.0 IBM, Armonk, NY, U.S.A.).

## Results

The total quantity of the phenolic and flavonoids content existing in the investigated extracts for each *Ficus carica* and *Olea europaea* was shown in Tables 1 and 2, which was found to be 8.576 ± 0.665 mg eq. gallic acid/g, 0.723 ± 0.042 mg eq. rutin/g, 13.457 ±1.245 and 0.998 ± 0.0689, respectively. Moreover, the results exposed that the both *Ficus carica* and *Olea europaea* have powerful free radical scavenging power. For the DPPH, ABTS, and FRAB assays, the values obtained were of 39.798 ± 1.99, 3.568 ± 0.025 0.102 ± 0.0004, 37.235 ± 1.98, 6.756 ± 0.0562, and 0.278 ± 0.0015 mol eq. Trolox/g, respectively.

**Table 1:** Determination of total quantity of the phenolic and flavonoids content existing in Fig (*Ficus carica*)

Parameters	Mean ± SD
Total phenols (mg eq. Gallic acid/g sample)	8.576 ± 0.665
Total flavonoids (mg eq. Rutin/g sample) DPPH (%)	0.723 ± 0.042
DPPH (%)	39.798 ± 1.99
ABTS (_mol eq. Trolox/g sample)	3.568 ± 0.025
FRAB (_mol eq. Trolox/g sample)	0.102 ± 0.0004

Experimental determinations of total phenolic and flavonoids contents and antioxidant capacity assays (ABTS, DPPH, and FRAB) for *Ficus carica*

**Table 2:** Determination of total quantity of the phenolic and flavonoids content existing in Olive (*Olea europaea*)

Parameters	Mean ± SD
Total phenols (mg eq. Gallic acid/g sample)	13.457 ±1.245
Total flavonoids (mg eq. Rutin/g sample) DPPH (%)	0.998 ± 0.0689
DPPH (%)	37.235 ± 1.98
ABTS (_mol eq. Trolox/g sample)	6.756 ± 0.0562
FRAB (_mol eq. Trolox/g sample)	0.278 ± 0.0015

Experimental determinations of total phenolic and flavonoids contents and antioxidant capacity assays (ABTS, DPPH, and FRAB) for *Olea europaea*

**Figure 1:**
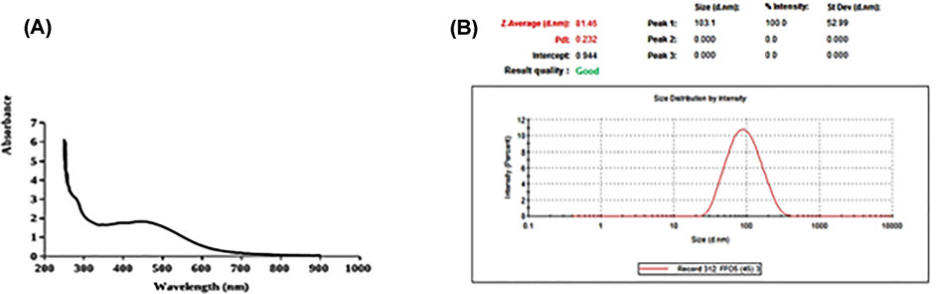
Determination of the synthesis of silver nanoparticles (**A**) The absorption spectrum of the (NFO). (**B**) Figure present a graph produced by using a zeta sizer for measuring the average size of (NFO).

**Figure 2:**
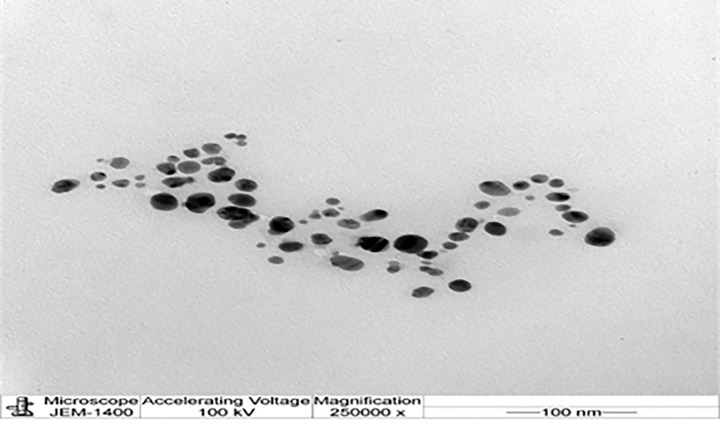
Determination of silver nanoparticles by transition electron microscopy (TEM) Figure presents a transition electron microscopy (TEM) image of (NFO) (scale bar: 100 nm).

A UV–visible spectrophotometer was used in order to confirm the presence of NFO. As shown in Figure 1A, the appearance of a band around 300 nm indicates the formation of NFO. In order to determine the size of the biosynthesized NFO particle, Zeta analysis was performed to determine the average particle diameter in nanometer (d.nm). NFO size distribution is shown in Figure 1B. The size of NFO particles ranged from 51 to 226 d.nm with an average size of 103.1 d.nm. Furthermore, TEM (Figure 2) demonstrated that most NFO were obviously spherical or polygonal in morphology, with a size ranging from 50 to 100 nm. Interestingly, after 50 days of storage at 4°C, the color of the NFO aqueous solution did not change indicating their stability. However, the color changed into colorless after 60 days.

**Figure 3:**
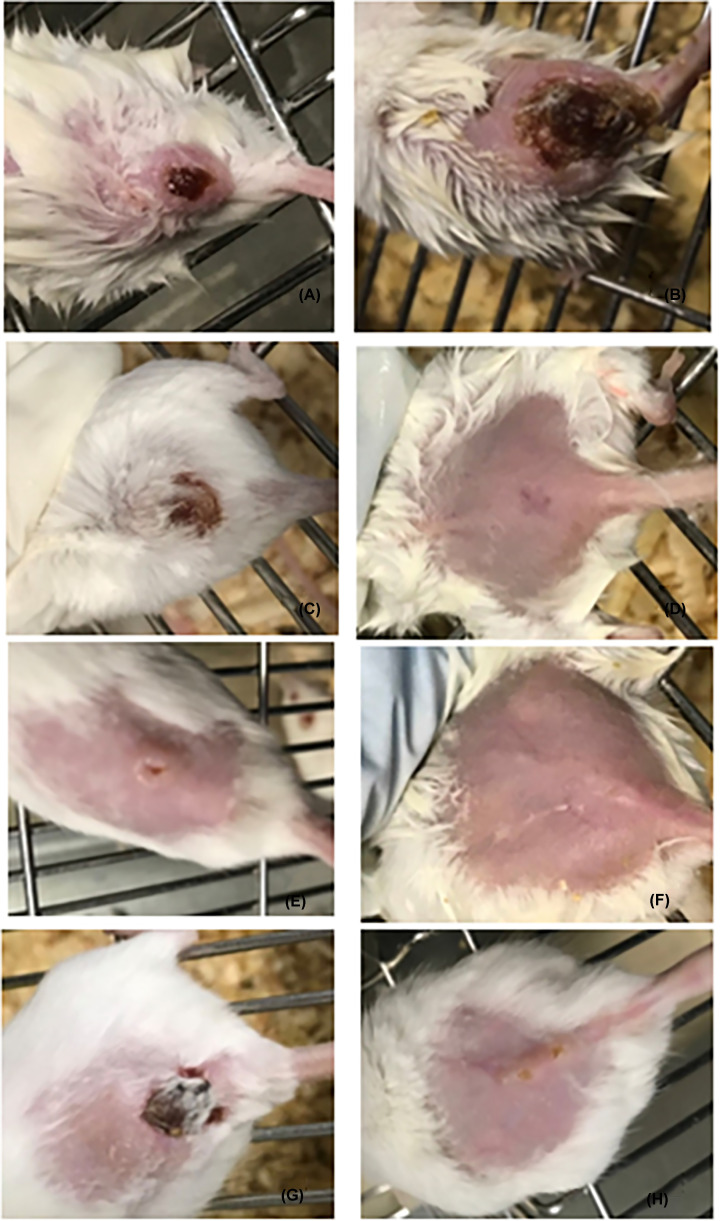
Lesion size of infected and treated mice CL lesions of infected mice (**A**) first week in (PC) mice showing redness, swelling, and ulcer formation. (**B**) Fourth week of (PC) mice showing redness, swelling, ulcer, and gangrene formation. (**C**) First week post-treatment with Pentostam showing redness, swelling, ulcer, and gangrene formation. (**D**) Fourth week post treatment with Pentostam showing complete recovery with scar formation. (**E**) First week post treatment with NFOA. (**F**) Third week post treatment with NFOA showing restored to normal skin. (**G**) First week post treatment with FOA. (**H**) 4^th^ week post treatment with FOA showing restored to normal skin with small ulcer.

Next, we examined whether concurrent treatment or pretreatment of mice with (NFO) could protect from *L. major* infection. Clinically, lesions in (PC) mice showed redness and swelling at the site of *L. major* inoculation at the fourth week post infection (Figure 3A,B).

**Figure 4:**
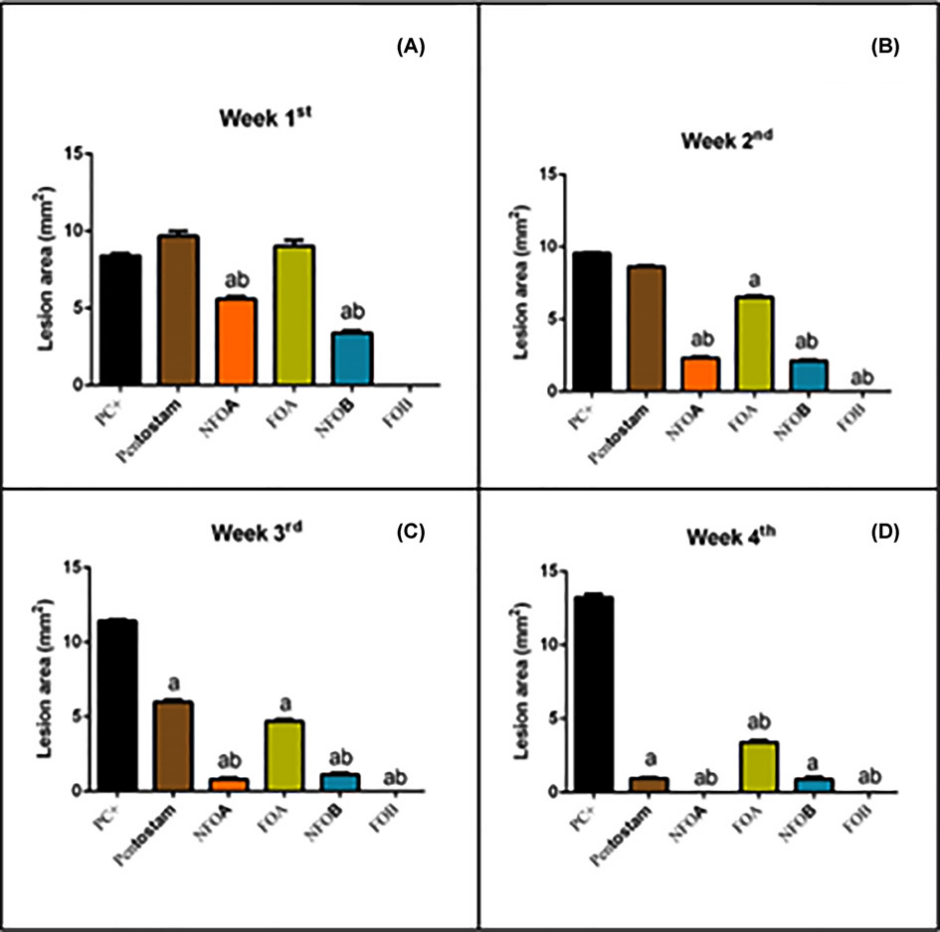
Determination CL lesion size CL Lesion size in mouse skin one (**A**), two (**B**), three (**C**), and four (**D**) weeks after infection. Lesion sizes measured with a digital caliper as described in the ‘Materials and Methods’ section. Each point represents the mean ± SD (*n*=10). Values are c ^a^*P*<0.05, indicates statistically significant changes compared with negative control (NC) group; ^b^*P*<0.05 indicates statistically significant changes compared with PC group.

**Figure 5:**
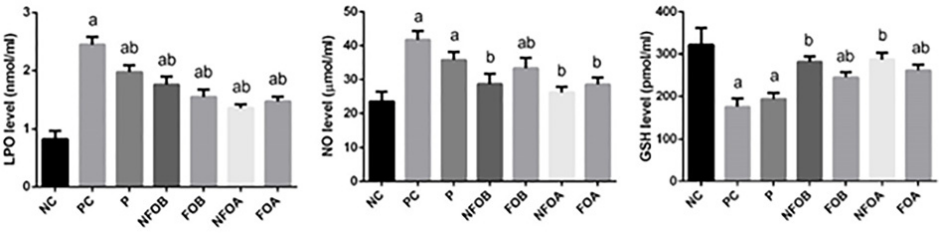
Determination of oxidative stress Effect of Ag-NPs biosynthesized by (Fig and Olive) extracts pretreatment or posttreatment and Pentostam on oxidative stress markers LPO, NO, and GSH of control and experimental groups 4 weeks after infection. Values are c ^a^*P*<0.05, indicates statistically significant changes compared with negative control (NC) group; ^b^*P*<0.05 indicates statistically significant changes compared with PC group. LPO, lipid peroxidation; NO, nitric oxide, and GSH, Glutathione.

In the infected control mice, the mean lesion size increased gradually by the fourth week following infection. It was observed that the lesion size started to decrease gradually after NFOA treatment was initiated and at the third week of treatment lesions had completely disappeared (Figure 4). CL infection significantly (*P*<0.05) enhanced lipid peroxidation LPO and nitric oxide NO production whereas, the dermal glutathione content was depleted significantly compared with the control mice. NFOA treatment significantly reversed the increase in LPO and NO, and the GSH (glutathione) content was increased significantly indicating the antioxidant property of NFOA (Figure 5).

**Figure 6:**
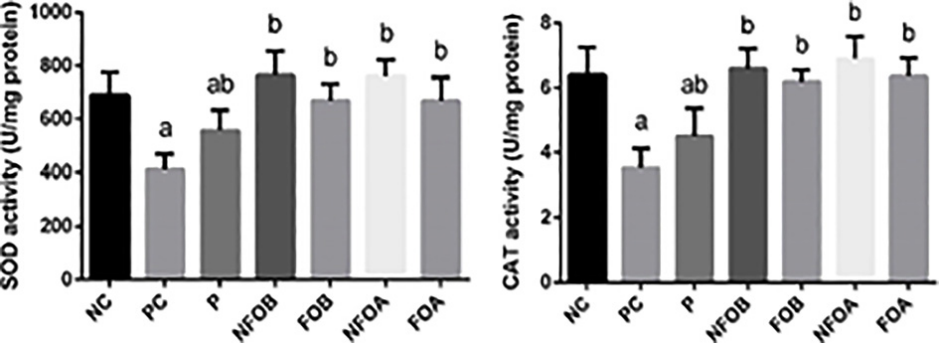
Determination enzymatic antioxidant status Effect of Ag-NPs biosynthesized by (Fig and Olive) extracts pretreatment or post-treatment and Pentostam on dermal antioxidant enzyme activities (SOD, CAT) of control and experimental groups four weeks after infection. Values are mean ± SD (*n*=10), ^a^*P*<0.05 indicates statistically significant changes compared with NC group; ^b^*P*<0.05 indicated statistically significant changes compared with PC group; SOD, superoxide dismutase; CAT, catalase.

Chronic wounds often exhibit oxidative stress that slows down the wound-healing process, suppresses collagen deposition and epithelialization and enzymatic antioxidants that play crucial roles in accelerating wound-healing [43]. We found that *L. major* infection of mice inhibited significantly (*P*<0.05) the antioxidant enzyme activities of superoxide dismutase (SOD) and catalase (CAT) (Figure 6). These biochemical findings were confirmed by the molecular analyses. NFOA treatment promoted the activity of the antioxidant enzymes and these enzymes showed higher activities than those in *L. major* infection mice. Furthermore, the results revealed that post treatment with NFOA led to better outcomes and Pentostam has no antioxidant activity.

**Figure 7:**
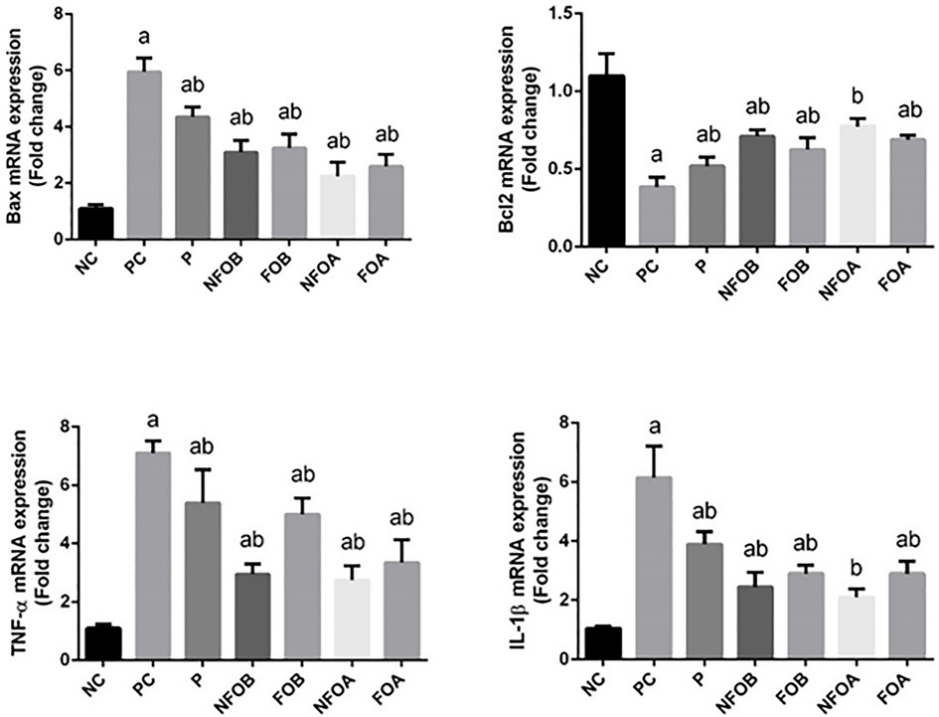
Gene epression profile by real-time PCR in skin Effect of Ag-NPs biosynthesized by (Fig and Olive) extracts pretreatment or post-treatment and Pentostam on dermal pro-apoptotic and anti-apoptotic (*BAX* and *BCL-2*) and pro-inflammatory cytokines (*TNF-α* and *IL-1β*) in control and experimental groups. Values are mean ± SD (*n*=10), ^a^*P*<0.05 indicates statistically significant changes compared with NC group; ^b^*P*<0.05 indicated statistically significant changes compared with PC group. BAX, BCL-2–associated X protein; BCL-2, B-cell lymphoma 2.

To investigate whether the observed anti-*leishmaniasis* effects of NFO were related to their anti-apoptotic activity, the protein levels of *BCL-2* and *BAX* in dermal tissue were measured. The expression levels of the anti-apoptotic protein *BCL-2* were significantly reduced (*P*<0.05), while those of the pro-apoptotic protein *BAX* were increased in *L. major* infected mice (Figure 7). However, mice treated with NFO concurrently or prior to *L. major* infection showed a significant increase in *BCL-2* and a significant decrease in *BAX*.

The present study also showed a significant up-regulation in the expression of *IL-1β* and *TNF-α* mRNA following infection with *L. major* compared with the control group. In contrast, treatment with NFO (pre. and post.) caused a significant decrease in the expression levels of *TNF-α* and *IL-1β* mRNAs (Figure 7).

**Figure 8:**
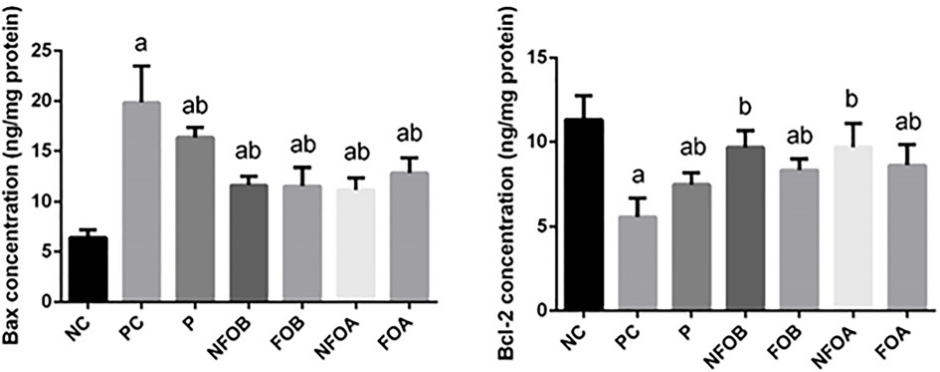
Determination of apoptotic markers in skin tissue Effect of Ag-NPs biosynthesized by Fig and Olive extracts pretreatment or post-treatment and Pentostam on dermal pro-apoptotic and anti-apoptotic proteins (Bax and Bcl-2) in control and experimental groups. Values are mean ± SD (*n*=10), ^a^*P*<0.05 indicates statistically significant changes compared with NC group; ^b^*P*<0.05 indicated statistically significant changes compared with PC group; BAX, BCL-2–associated X protein, BCL-2, B-cell lymphoma 2.

To investigate whether the observed anti-*leishmaniasis* effects of NFO were related to the anti-apoptotic activity of NFO, the protein levels of Bcl-2 and Bax in dermal tissue were measured. The current findings revealed that the anti-apoptotic protein Bcl-2 was significantly reduced (*P*<0.05), while the pro-apoptotic protein Bax was increased in *L. major* infection mice (Figure 8). However, mice treated with NFO concurrently or prior to *L. major* infection showed significant increase of Bcl-2 and decreased significantly the Bax level.

**Figure 9:**
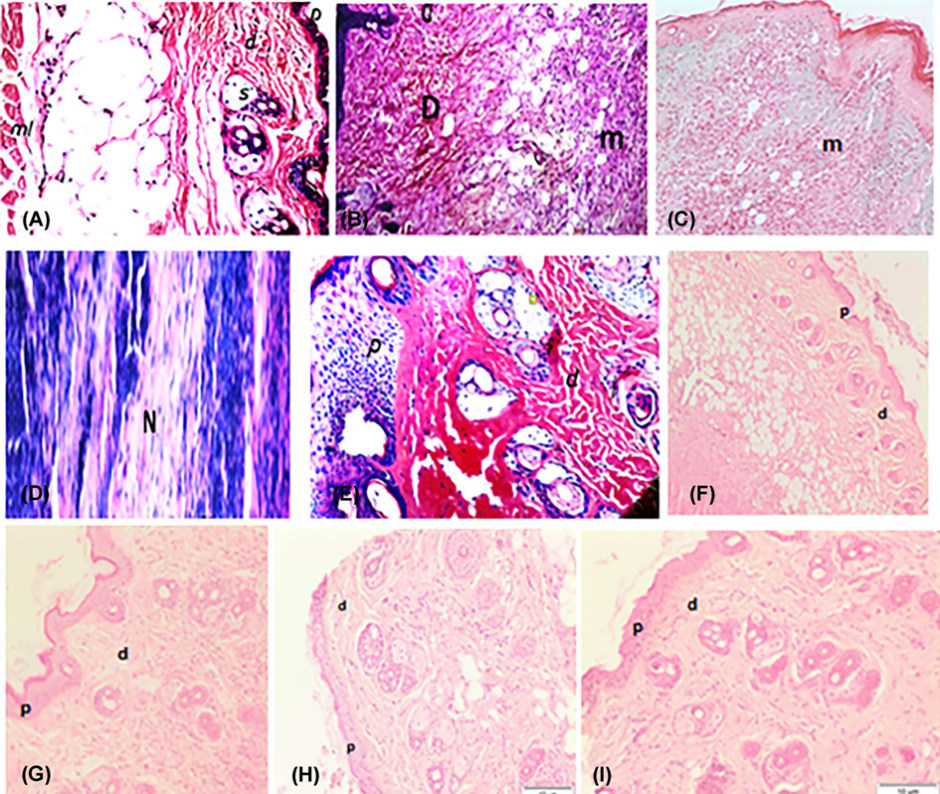
Examination of the histological changes in the skin of mice Hematoxylin and eosin-stained skin sections. (**A**) Control animals showed no abnormalities, no histopathological alteration and normal histological structure of the epidermis, dermis, subcutaneous, and musculature tissue. (**B** and** C**) Skin section of the PC group at 4 weeks post-infection showing highly inflammatory cells infiltration in diffuse manner in subcutaneous tissue (m). (**D** and **E**) Pentostam treated mice at 4-week post-treatment showing acanthosis in the epidermis (P), while the underlying deep dermis and subcutaneous musculature showed inflammatory cells infiltration. No histopathological alteration in both epidermal and dermal layers. (**E**) Skin of mice showing suppurative liquefactive necrosis in deep subcutaneous tissue (N). (**F**) Mice treated orally with (NFOA) at the third week post-treatment, showing an intact epidermis (p) and dermis (d) with remarkable reduction in the inflammatory response. (**G**) Mice received orally treatment (NFOB) two weeks before infection, showing normal histological structure of epidermis (P) and dermis (**D**) and few inflammatory cells infiltration in dermal and subcutaneous tissue. (**H**) Mice treated only with (FOA) showed normal histological structure of epidermis and dermis and few inflammatory cells infiltration in dermal and subcutaneous tissue. (**I**) Mice received (FOB) daily 2 weeks before infection, showing normal histological structure of epidermis and dermis and few inflammatory cells infiltration in dermal and subcutaneous tissue.

Examination of the histological changes in the skin of mice from all the groups supported the results observed from the previous experiments (Figure 9). Four weeks after infection, the skin of infected mice showed a moderately dense, localized dermal infiltrate composed of mixed acute and chronic nonspecific inflammatory cellular infiltrates. Congested dilated blood vessels were observed, and numerous *Leishmania* promastigotes were seen either inside or outside the macrophages. Moreover, suppurative liquefactive necrosis was observed in deep subcutaneous tissue. In Pentostam-treated mice, the skin showed normal histological structure of subcutaneous musculature tissue and infiltration of few inflammatory cells in the subcutaneous tissue. However, apparent ameliorations were noticed. In the NFOA group, the tissue sections showed an intact epidermis with a moderately dense infiltrate, milder grade of infection in terms of both the inflammatory response and the number of visible amastigotes. Furthermore, the intensity of infection was also less than that of the infected untreated controls. Surprisingly, no sign of pathological changes was found in mice NFOB pretreated and those treated with NFOA.

## Discussion

Green synthesis nanoparticles are a great interest, since their large-scale application in the biomedical sector (nanomedicine). This is due to synthesized by green technologies in the size range from 1 to 100 nanometers exhibit antioxidant, anti-inflammatory, and immunomodulatory activities. A distinctive feature of the nanoparticle’s synthesis with use plants called (phytosynthesis) due to a higher rate of nanoparticle formation and contains a wide range of biomolecules such as, (poly) phenolic and flavonoids compounds [44,45].

In the present study, the plants species of Figs and Olives were as a source for the synthesis of Ag-NPs. The formation of NFO was confirmed by the change in color and the stability in the solution. TEM analysis determined that the range of particle size was about 100 nm and that they were spherical in shape. The flavonoids, polyphenols, and other constituents present in the extracts of Figs and Olives act as the surface-active stabilizing molecules for the synthesis of (Ag-NPs) with antioxidative and anti-inflammatory activities [46].

In the present study, we demonstrate that *Ficus carica Linn* (Fig) and *Olea europaea* (Olive) extracts with (Ag-NPs) improved skin lesions and promote their healing and parasite resolution in Balb/c mice infected with *L. major*. This activity may be attributed to the presence of phenolic and flavonoids compounds that possesses antileishmanial potency by enhancing nonspecific immunity by macrophages activation and inducing NO, interferon-gamma and tumor necrosis factor-gamma, so those chemicals and cytokines produce fundamental host defense system and kill the invading parasite [47,48]

NFOA treatment caused a significant reduction in the average lesion size and complete healing after 21 days compared with standard drug Pentostam that needs more than 28 days for healing. This is in accordance with previous reports [49–53].

Reactive oxygen species (ROS) formed during multiple normal processes in tissues and cells are involved in the pathogenesis of various parasitic infections including *Leishmania spp*., *Toxoplasma gondii, Giardia lamblia*, and *Entameba histolytica* [54–57]. *Leishmania L. major* induce inflammation by mast cell stimulation and by secreting proinflammatory mediators. ROS that are produced during an inflammatory response lead to oxidative injury to noninfected cells. During oxidative damage, released free radicals play an important role in collagen damage [58–61].

The overproduction of NO levels in response to parasitic infection may be considered as one of the factors inducing oxidative stress and inflecting tissue injury [62]. Finally, NO production correlates positively with tissue fibrosis through inducing fibrogenic cytokines and increasing collagen synthesis [63–65].

In our study, CL infection impaired the antioxidant system, GSH is the main endogenous antioxidants, was depleted and associated with lipid peroxidation, a marker of cellular oxidative stress. In the present study, an elevation in MDA, the product of LPO in infected and Pentostam groups, was observed. LPO has been traditionally thought to be the major effect of free radicals. It leads to impairment in the physicochemical properties of the membrane including its fluidity and integrity and to the induction of apoptosis [66]. Treatment with NFOA increased the GSH content [64], and subsequently reduced the formation of intracellular ROS in response to different pro-oxidant stimuli [67]. The present data suggest that NFOB is capable of protecting cells by stabilizing the membrane permeability through inhibiting LPO and preventing GSH depletion.

The activities of antioxidant enzymes SOD and CAT in the skin tissue of mice infected with CL also decreased; CAT detoxifies hydrogen to water while SOD catalyzes the reduction of superoxide anions to hydrogen peroxide [68].

To explore the mechanism by which NFO attenuate CL-induced apoptosis, the levels of BAX and BCL-2 protein were measured in skin homogenates. ROS have been shown to increase the permeability of the mitochondrial membrane and to result in mitochondrial failure [69–72]. The permeability of the mitochondrial membrane is dependent upon the mitochondrial permeability transition pore that mediates the release of cytochrome *c* from the mitochondria to the cytosol [73]. Once released, cytochrome *c* can bind to apoptotic protease-activating factor-1 (Apaf-1) in the cytoplasm forming a complex that can activate caspase-9 with subsequent induction of death [66,74,75]. The mitochondria-mediated intrinsic apoptotic pathway is controlled by BCL-2 family proteins. The BCL-2 protein family is classified into two subgroups according to structural homology: the anti-apoptotic proteins such as BCL-2, BCL-XL, and the pro-apoptotic proteins such as BAX and Bak. The balance between the pro- and anti-apoptotic proteins of the BCL-2 family is important to determine cell survival or death. BCL-2 has also been found to function as a counteracting force to reduce the damage mediated by LPO triggered by cytotoxic stimuli such as ROS [76,77]. BCL-2 was also found to prevent the release of cytochrome *c*. In contrast, BAX regulates apoptosis, not only by dimerizing with anti-apoptotic BCL-2 proteins, but also by regulating cytochrome c release and subsequent caspase-3 activation [78–80]. The present study also showed that treatment of mice with NFO reversed the CL-induced alternations in BCL-2 and BAX levels, and substantially restored the ratio of BCL-2/BAX. In the current report, NFO inhibited all toxic events induced by CL. It is known that extracts with silver nanoparticles scavenge oxygen and nitrogen reactive species generated in mitochondria, stabilize the mitochondrial membrane and enhance anti-apoptotic signaling.

Inflammation is characterized by the release of proinflammatory cytokines such as TNF-α, IL-1β, and IL-6, as well as inflammatory mediators, including nitric oxide (NO) and prostaglandin E2 (PGE2), which are synthesized by inducible nitric oxide synthase (iNOS) and cyclooxygenase (COX). These inflammatory mediators and cytokines are involved in the causation of many human diseases including rheumatoid arthritis, asthma, atherosclerosis, infections, and endotoxin-induced multiple organ injury [81–83]. Anti-inflammatory agents reduce the inflammatory response by suppressing the production of inflammatory cytokines and mediators [84,85].

During infection, the inflammation is a response by macrophages to effect healing and restoration. The kinetics of the responses of proinflammatory, anti-inflammatory cytokines, and inflammatory master regulator nuclear factor-κB (NF-κB) elicited by pathogen-associated molecular patterns, such as lipopolysaccharide (LPS), and virulence factors are microbial inducers of inflammation which is may be critical determinants of the inflammatory response by macrophages [86].

The production of these cytokines typically proceeds via host cell signaling cascades following the engagement of innate pathogen-associated molecular pattern (PAMP) receptors including the Toll-like receptors (TLRs) expressed primarily by cells of the innate immune compartment and by pathogen-specific ligands, such as LPS [87].

The exposure of macrophages to *Leishmania* leads to the generation of ROS, which contribute to the regulation of the inflammatory response controlled by the cellular antioxidant defense system [88,89].

NF-κB has a seminal role in immunity, because it induced the expression of pro-inflammatory genes encoding *iNOS, COX-2, TNF-α, IL-1β*, and *IL-6* [85,90]. It is activated by phosphorylation, ubiquitination, and subsequent proteolytic degradation of the IκB protein by activated IκB kinase (IKK) [91]. The liberated NF-κB translocates to the nucleus and binds as a transcription factor to κB motifs in the promoters of target genes, leading to their transcription. Aberrant NF-κB activity is associated with various inflammatory diseases, and most anti-inflammatory drugs suppress inflammatory cytokine expression by inhibiting the NF-κB pathway [92,93]. Thus, an NF-κB inhibitor has clinical potential in inflammatory diseases.

The lipopolysaccharides receptor, LPS, interacts with the small GTP-binding protein Rac1, and then associates with p47phox and p67phox (two subunits needed for NADPH oxidase p91phox function) at the cell membrane, leading to ROS production [94].

Our findings demonstrate that the natural product nanoparticles inhibited signaling cascade of proinflammatory gene expression in LPS-stimulated macrophages by suppressing NF-κB activation, probably as a result of scavenging intracellular ROS. This inhibitory effect has important implications for the development of anti-inflammatory drugs and strategies to limit pathological inflammation [95–97].

The present study showed a significant up-regulation in the *TNF-α, IL-1β*, and *BAX* genes expression levels caused by *leishmania* infection, while *BCL*-2 gene was up-regulated in mice treated with NFOA compared with the control group. In contrast with our data, it has been reported that nanoparticles cause a significant decrease in the expression of *TNF-α, IL-1β*, and *BAX* genes. This is in agreement with previous reports [98,99].

## Conclusions

These findings indicated that Ag-NPs biosynthesized from Fig and Olive extracts could be considered as a safe chemotherapeutic agent, for fast treatment of *Leishmaniasis* and complete healing after 21 days compared with standard drug Pentostam (needs more than 28 days for healing). It could be a possible new anti-leishmanial drug against *Leishmania major*.

## Abbreviations

BAX: BCL-2–associated X protein
BCL-2: B-cell lymphoma 2
COX: cyclooxygenase
iNOS: inducible nitric oxide synthase
NO: nitric oxide
PAMP: pathogen-associated molecular pattern
PGE2: prostaglandin E2
ROS: reactive oxygen species
SOD: superoxide dismutase
TLR: Toll-like receptor
